# Characterisation of a *C1qtnf5* Ser163Arg Knock-In Mouse Model of Late-Onset Retinal Macular Degeneration

**DOI:** 10.1371/journal.pone.0027433

**Published:** 2011-11-16

**Authors:** Xinhua Shu, Ulrich F. O. Luhmann, Tomas S. Aleman, Susan E. Barker, Alan Lennon, Brian Tulloch, Mei Chen, Heping Xu, Samuel G. Jacobson, Robin Ali, Alan F. Wright

**Affiliations:** 1 MRC Human Genetics Unit, Institute of Genetics and Molecular Medicine, Edinburgh, United Kingdom; 2 UCL Institute of Ophthalmology, London, United Kingdom; 3 Department of Biological and Biomedical Sciences, Glasgow Caledonian University, Glasgow, United Kingdom; 4 Department of Vision Sciences, Glasgow Caledonian University, Glasgow, United Kingdom; 5 Scheie Eye Institute, University of Pennsylvania School of Medicine, Philadelphia, Pennsylvania, United States of America; 6 Centre for Vision and Vascular Science, Queen's University Belfast, Belfast, United Kingdom; Faculty of Medicine University of Leipzig, Germany

## Abstract

A single founder mutation resulting in a Ser163Arg substitution in the *C1QTNF5* gene product causes autosomal dominant late-onset retinal macular degeneration (L-ORMD) in humans, which has clinical and pathological features resembling age-related macular degeneration. We generated and characterised a mouse “knock-in” model carrying the Ser163Arg mutation in the orthologous murine *C1qtnf5* gene by site-directed mutagenesis and homologous recombination into mouse embryonic stem cells. Biochemical, immunological, electron microscopic, fundus autofluorescence, electroretinography and laser photocoagulation analyses were used to characterise the mouse model. Heterozygous and homozygous knock-in mice showed no significant abnormality in any of the above measures at time points up to 2 years. This result contrasts with another *C1qtnf5* Ser163Arg knock-in mouse which showed most of the features of L-ORMD but differed in genetic background and targeting construct.

## Introduction

Late-onset retinal macular degeneration (L-ORMD) is a fully penetrant autosomal dominant disorder associated with a late-onset macular degeneration resembling age-related macular degeneration (AMD) [1], [2], [3]. L-ORMD shows disease onset in the fifth to sixth decade with impaired dark adaptation associated with both punctate and diffuse sub-retinal pigment epithelium (RPE) deposits, leading to central and later peripheral visual loss and, at late stages, a pan-retinal atrophy often with choroidal neovascularisation (CNV) and disciform scarring. The most striking and consistent pathological feature is a thick (≤50 µm) extracellular sub-RPE deposit, worst in the macula but extending to the extreme retinal periphery [2], [3]. The deposits resemble basal laminar deposits that can be seen both in aged and in AMD eyes, with wide-spaced collagen, RPE basal processes penetrating the deposits and appearances consistent with exocytosis of packets of fibrillar material into the deposits [3]. An unusual phenotypic feature is the presence of long ciliary zonules, which extend from the ciliary epithelium to the anterior lens [4], [5].

L-ORMD is caused by a single founder Ser163Arg mutation in the Complement 1q Tumour Necrosis Factor 5 (*C1QTNF5*) gene [1]. *C1QTNF5* (formerly called *CTRP5*) encodes a short-chain collagen which is strongly expressed in RPE, ciliary epithelium and adipose tissue [1], [4], [6]. The protein is predicted to contain an N-terminal secretory signal, a short helical collagen repeat and a C-terminal globular complement 1q (gC1q) domain which facilitates trimerisation [1], [7], [8]. The functional consequences of a Ser163Arg mutation in the gC1q domain has been reported to involve destabilisation of the protein as a result of an abnormal surface charge [7], [8]. Similar to the closely-related collagens VIII and X, C1QTNF5 appears to be secreted into the adjacent extracellular matrix but its function is currently unknown. C1QTNF5 protein interacts with the CUB domains of the Membrane type Frizzled Related Protein (*MFRP*) [8]. *MFRP* is expressed as a dicistronic transcript with *C1QTNF5* [1] and is mutated in human autosomal recessive nanophthalmos and also in the mouse, in the *Mfrp rd6* mutant, which is associated with retinal degeneration [9], [10]. Recently, Park *et al.* reported that the expression of C1QTNF5 is increased in mtDNA-depleted myocytes and that it stimulates the phosphorylation of AMP-activated protein kinase [11]. These authors also showed that serum C1QTNF5 has significantly higher expression in obese/diabetic mice than in controls.

In order to investigate the pathogenic role of the *C1QTNF5* Ser163Arg mutation *in vivo*, we generated a *C1qtnf5* Ser163Arg knock-in mouse model of L-ORMD by homologous recombination into mouse embryonic stem cells and analysed the consequences of the mutation on retinal function and morphology.

## Results

### Generation of *C1qtnf5* Ser163Arg mice

**Figure 1:**
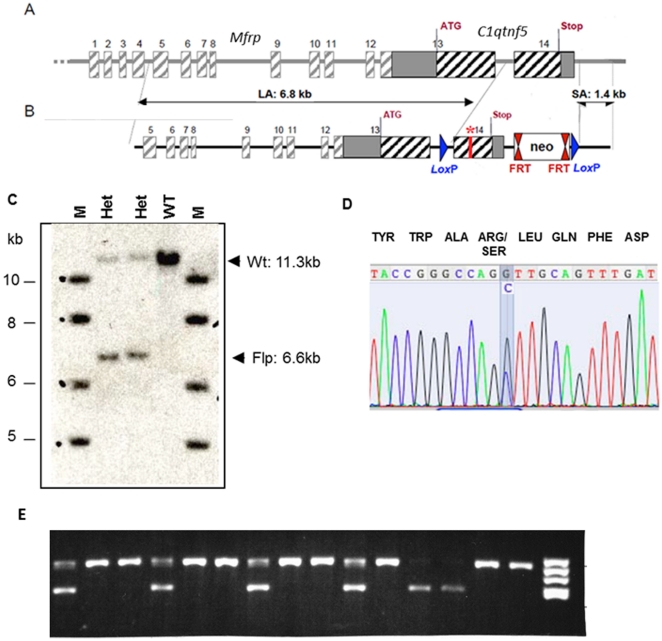
Introduction of the Ser163Arg mutation into the mouse *C1qtnf5* gene. A) Schematic representation of the murine *Mfrp*/*C1qtnf5* genes. Boxes represent exons, the solid line represents intronic sequence (not drawn to scale). B) The targeting construct showing the long (6.8 kb) homology arm (LA), short (1.4 kb) homology arm (SA) and the central fragment with the Ser163Arg mutation labelled with a *. FRT: Flippase Recognition Target sites, Neo: the neomycin selection cassette. LoxP: sites flanking the introduced mutation and Neo gene, allowing subsequent Cre-recombinase-mediated deletion to generate a knockout mouse. C) Southern blot performed using genomic DNA from two heterozygous mice with a 3′ *C1qtnf5* probe showing wild-type genomic DNA digested by AvrII, resulting in a 11.3-kb band, while genomic DNA containing the targeted Ser163Arg mutant showed the expected 6.6-kb band following Flp-mediated excision of the neo cassette. D) Validation of the Ser163Arg point mutation in heterozygous mice by DNA sequencing. The wild-type codon is AGC (serine), the mutant is AGG (arginine), the heterozygous mice show both alleles, highlighted in blue. E) Genotyping of tail biopsy DNA from wild-type and mutant (*C1qtnf5* Ser163Arg) mice by PCR amplification of the native wild-type and mutant fragments. The primers anneal close to the FRT-sites flanking the neo cassette (see Figure 1 and Materials and Methods). The wild-type allele corresponds to the lower 432 bp band and the mutant allele to the upper 548 bp band. The gel shows genotypes in a mixture of *C1qtnf5* Ser163Arg homozygous mutant (n = 9), heterozygous mutant (n = 4) and wild-type mice (n = 2). A DNA molecular weight marker V (8–587 bp; *Hae*III digested pBR322 (Roche)) is shown in the right hand lane.

Both human C1QTNF5 and mouse C1qtnf5 proteins contain 243 amino acids with 94% identity. In humans, the Ser163Arg mutation is caused by a single point mutation in codon 163 (AGC>AGG) changing the encoded serine to an arginine residue. In the mouse, serine is also encoded by an AGC codon, therefore the same point mutation (AGC>AGG) in mouse *C1qtnf5* introduces the mutation found in L-ORMD patients. The targeting strategy and *C1qtnf5* Ser163Arg targeting construct are described in detail in Materials and Methods and summarised in Figure 1. The targeting vector contained long (6.8 kb) and short (1.4 kb) genomic fragments from the *Mfrp*/*C1qtnf5* locus together with a neomycin resistance (neo) cassette flanked by flippase (Flp) recombination target (FRT) sites in order to remove the neo cassette following successful targeting (Figure 1B). LoxP sites were also introduced, which can be used in the future for deleting the second and final exon of *C1qtnf5*, creating a *C1qtnf5* null mouse. The linearized construct was electroporated into mouse 129SV embryonic stem (ES) cells and 271 G418 (neo) resistant clones were isolated. These were initially screened by polymerase chain reaction (PCR) amplification, which identified 10 potentially targeted clones, which were further characterised by PCR, Southern blotting and sequencing (data not shown). Four ES cell clones with the *C1qtnf5* Ser163Arg neo allele present were fully validated and 3 of these were injected into C57BL/6J mouse blastocysts to generate chimaeric mice. Two highly chimaeric males (with 85% and 98% chimaerism) were each mated with two Flp recombinase deleter C57BL/6J females to remove the neo cassette. Two mice (one male and one female) were found to be mosaic for the Ser163Arg mutation in the F1 progeny. The two mosaic mice were each mated with wild-type mice, which gave rise to 15 pups. The 15 animals were screened by PCR to determine whether complete excision of the neomycin cassette had occurred at the targeted *C1qtnf5* locus. Five animals were heterozygous for the *C1qtnf5* Ser163Arg mutation and were validated by Southern blotting and sequencing (Figure 1C, D). Intercrossing of heterozygous *C1qtnf5* ^+/Ser163Arg^ mice on a 129SV background generated wild-type, heterozygous *C1qtnf5* ^+/Ser163Arg^ and homozygous C1qtnf5^Ser163Arg/Ser163Arg^ mice. Both heterozygous (*C1qtnf5* ^+/Ser163Arg^) and homozygous (*C1qtnf5* ^Ser163Arg/Ser163Arg^) mice were fertile, with normal weight and lifespan, and did not show evidence of systemic disease.

**Figure 2:**
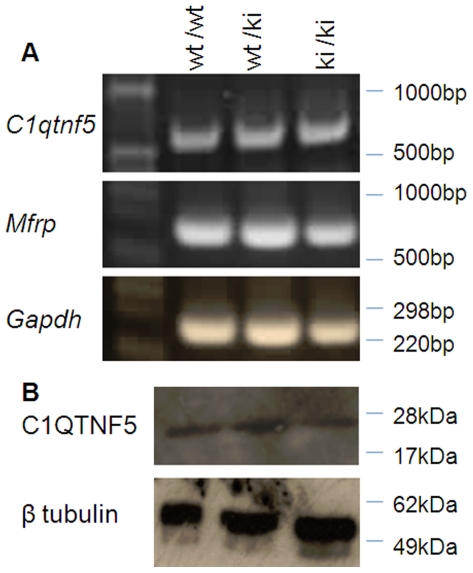
*C1qtnf5* and *Mfrp* expression in *C1qtnf5* Ser163Arg knock-in (KI) mice. A) Similar expression of *C1qtnf5* and *Mfrp* by reverse transcriptase-PCR in RPE/choroid from wild-type (wt) and heterozygous (wt/ki) or homozygous (ki/ki) *C1qtnf5* Ser163Arg KI mice. B) Western blot of eye cups from wild-type and *C1qtnf5* heterozygous and homozygous knock-in mice stained with anti-C1QTNF5 antibody (top) and anti-β tubulin antibody (bottom).

The mRNA from the mutant *C1qtnf5* Ser163Arg allele was found by reverse transcriptase PCR (RT-PCR) to be expressed at similar levels to the wild-type allele, showing that the introduction of the mutant allele did not affect its expression (Figure 2A). The expression of *Mfrp*, which is co-ordinately expressed as a dicistronic partner with *C1qtnf5*, was also unaltered (Figure 2A). C1qtnf5 protein levels in the eyes of both heterozygous and homozygous *C1qtnf5* Ser163Arg knock-in (KI) mice were also similar to wild-type (Figure 2B).

### Phenotypic analysis of the *C1qtnf5* Ser163Arg mice

**Figure 3:**
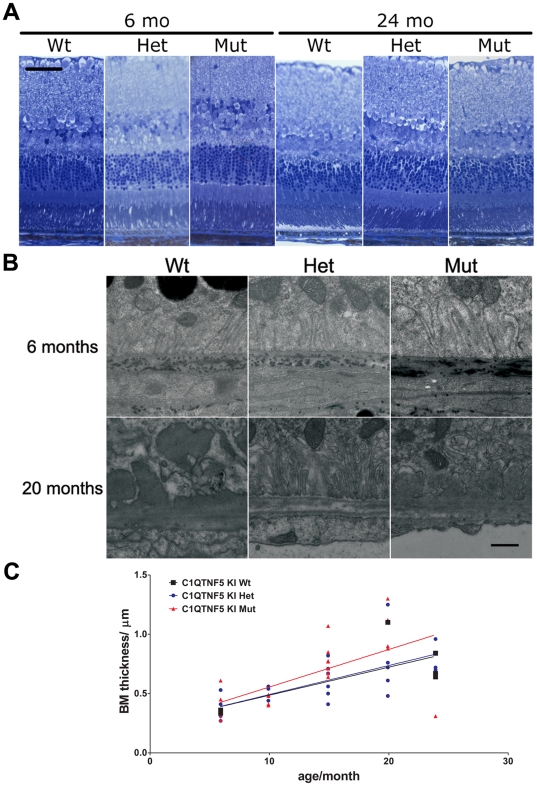
Retinal structure and ultrastructure in *C1qtnf5* knock-in (KI) and wild-type (Wt) mice. A) Retinal sections from mice of the *C1qtnf5* wild-type, Ser163 Arg heterozygous (Het) and homozygous (Mut) genotypes at the ages indicated, stained and examined by light microscopy. B) Ultrastructures of the basal site of the RPE and Bruch's membrane of retinas from wild-type (Wt) and *C1qtnf5* Ser163Arg KI (Het, Mut) mice at the ages of 6 months and 20 months, respectively. C) The thickness of Bruch's membrane increased with age (Pearson correlation Wt: n = 6, p = 0.103, r^2^ = 0.526; Het: n = 22, p = 0.0005, r^2^ = 0.459; Mut: n = 21, p = 0.0039, r^2^ = 0.362) but no significant difference between wild-type and *C1qtnf5* Ser163Arg KI mice was found.

A series of experiments were carried out to characterise the ocular phenotype of the *C1qtnf5* Ser163Arg mice. Histological evaluation by light microscopy and electron microscopy showed the neural retina of *C1qtnf5* heterozygous and homozygous KI mice to be essentially normal from age 6 to 24 months of age, compared with those of age-matched wild-type mice (summarised in Figure 3A). We evaluated the integrity of the RPE and the thickness of Bruch's membrane (BM) in young (6 months) and old (20 months) wild-type and Ser163Arg KI mice and focused in particular on age-related changes in the retinal pigment epithelium (RPE) and underlying BM. Although aged animals showed amorphous sub-RPE deposits, there was no obvious difference in these deposits between wild-type and Ser163Arg KI mice (Figure 3B). The BM thickness increased with age, so there was a significant correlation between age and BM thickness in each genotype respectively. The values of mean BM thickness of old mice (20–24 months) were approximately twice those from young animals (6 months), but we did not identify a significant difference between age-matched wild-type and Ser163Arg KI mice (Figure 3B, C), indicating that the major cause of the increase in BM thickness was normal ageing.

**Figure 4:**
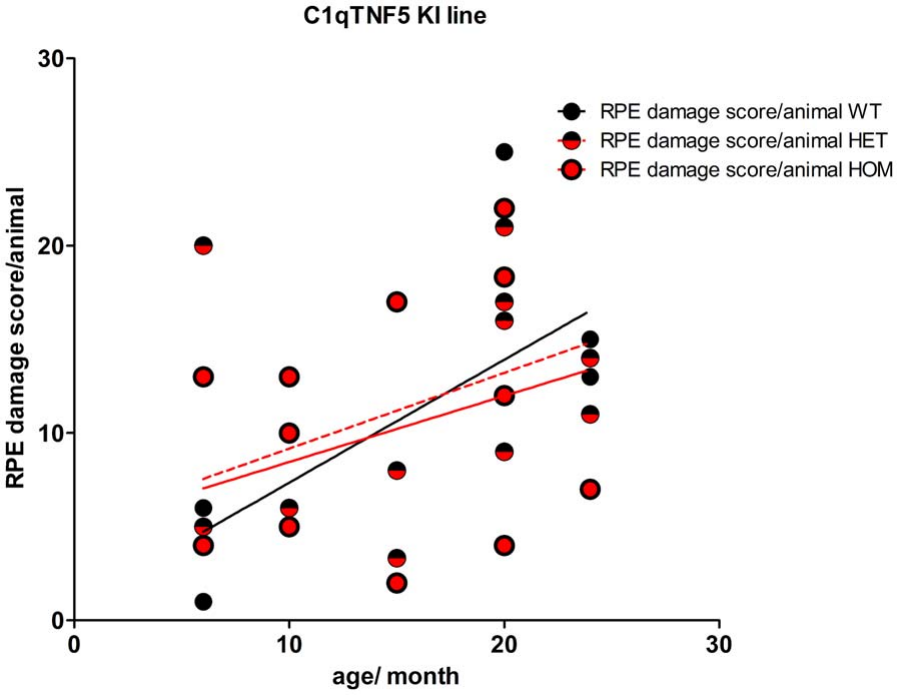
Retinal pigment epithelium (RPE) damage scores in *C1qtnf5* wild-type and Ser163 Arg mutant mice. RPE damage was scored in wild-type and *C1qtnf5* Ser163Arg knock-in (KI) mice at ages between 6 and 24 months old and no signficant difference was found between any of the genotypes (n (wt) = 6, n(het) = 15, n(mut) = 14). Overall, there is a signficant correlation of RPE damage with age (Pearson correlation n(wt,het,mut) = 35, p = 0.0026, r^2^ = 0.244).

To assess any possible differences in age-related RPE pathology between wild-type, heterozygous or homozygous mutant *C1qtnf5* KI littermates with age, we applied a previously described method for RPE pathology grading in animals between 6 and 24 months of age [12], [13]. In Figure 4, the mean sum of the observed RPE damage per section and per animal is shown, estimated from the analysis of three sections per animal. All three genotype groups showed a similar range of RPE damage, that was not significantly different between the three groups (Pearson correlation, p = 0.0026, N = 35 r^2^ = 0.244). This suggests that the observed RPE damage increases with age in all three groups and so is most likely to be due to normal ageing and not to an effect of the Ser163Arg KI allele. These findings are very similar to those in a previous study, in which we also showed a significant increase of RPE damage with age in wild-type mice over a period of 2 to 24 months [13].

**Figure 5:**
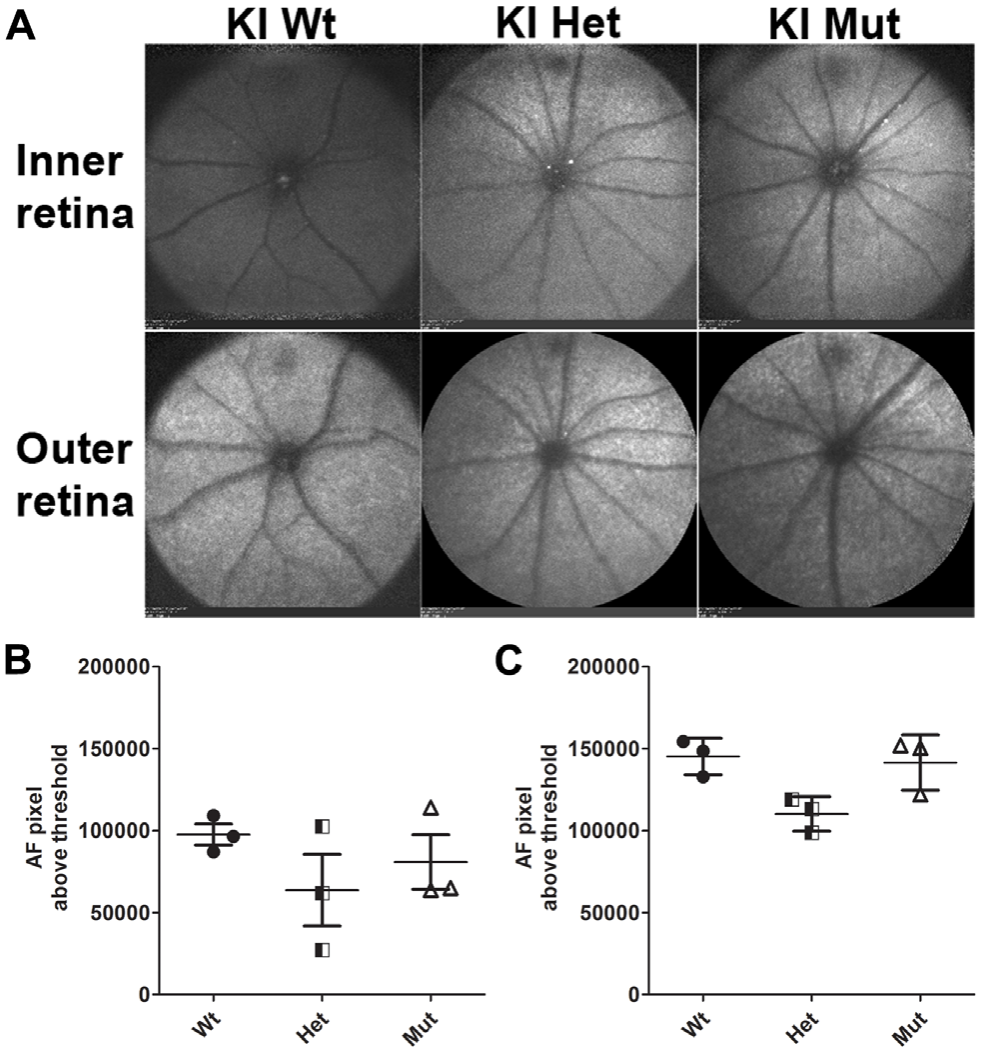
Fundus autofluorescence (AF) in *C1qtnf5* wild-type and Ser163Arg knock-in mice. A) Representative fundus images of inner and outer retina obtained by AF-SLO imaging from wild-type, heterozygous and homozygous mutant *C1qtnf5* S163 KI mice at 16–18 months of age. No obvious difference in clearly demarcated autofluorescent areas or punctuated pattterns in the fundus images were observed in any of the three gentotypes. Wt: wild-type mice, Het: heterozygous KI mice, Mut: homozygous KI mice. B,C) Semiquantitative analysis of the inner (B) and outer (C) retinal background autofluorescence. The mean background autofluorescence (AF-pixel above threshold) for the right and left eye per animal is shown for each genotype (n = 3 animals per genotype). No significant difference in background autofluorescence in the inner retina (B, Kruskal-Wallis with Dunn's multiple comparison test, p = 0.393) or in the outer retina (C, Kruskal-Wallis with Dunn's multiple comparison test, p = 0.067) was observed between any of the three gentoytpes.

Analysis of changes in the normal distribution of fluorophores inside the retina and RPE was performed using autofluorescence-scanning laser ophthalmoscopy (AF-SLO) in three wild-type, *C1qtnf5* ^+/Ser163Arg^ and *C1qtnf5* ^Ser163Arg/Ser163Arg^ KI mice at 15–16 months of age respectively. There was no significant difference in autofluorescence in inner and outer retina between wild-type and *C1qtnf5* Ser163Arg KI mice, although the number of autofluorescence spots in the inner and outer retina of *C1qtnf5* ^Ser163Arg/Ser163Arg^ mice were slightly greater than those of wild-type mice but this was not statistically significant (Figure 5).

**Figure 6:**
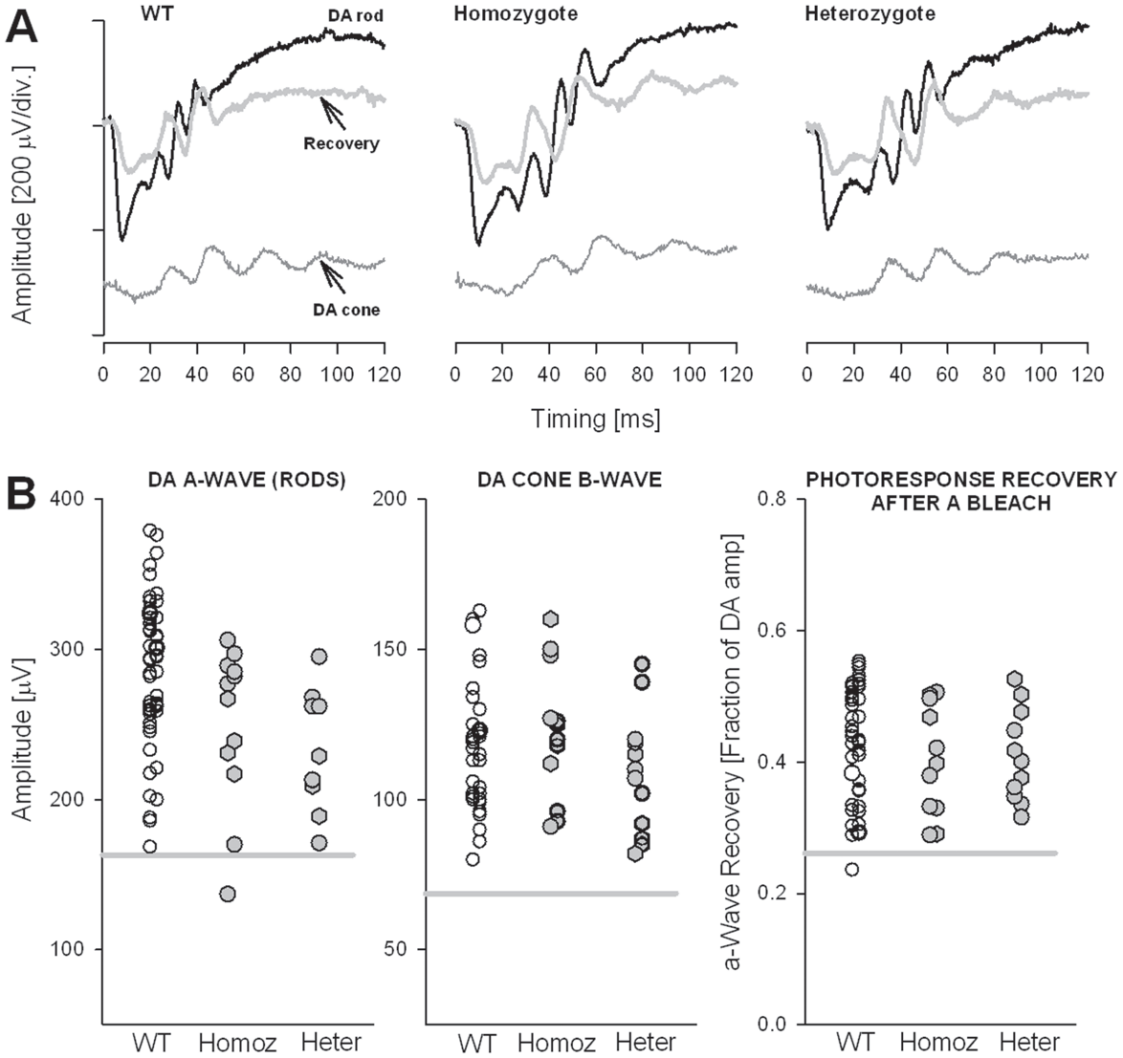
Electroretinography in wild-type and *C1qtnf5* Ser163Arg knock-in mice. **A**, Dark-adapted (DA) ERGs evoked by 2.2 log scot-cd.s.m^−2^ flashes (black traces) in 19-month-old heterozygote and homozygote, *C1qtnf5* Ser163Arg knock-in mice compared to responses from a representative, wild-type (WT) control. DA cone function (bottom grey traces) was isolated in dark-adapted mice by recording ERGs within a short interval following a rod-suppressing bleaching light; recovery of the rod-mediated ERG response (grey traces overlapping DA rod traces) was assessed by recording ERGs 10 minutes following this bleach. **B**, Summary statistics of ERG parameters, showing a-wave (rods, left panel) and b-wave (cones, middle panel) in wild-type (WT), *C1qtnf5* Ser163Arg heterozygous (Heter) and homozygous (Homoz) knock-in mice. Recovery of the a-wave (photoresponse) after the bleaching exposure is shown (right panel) and expressed as a fraction of the dark-adapted amplitude. The results for both eyes (right eye symbols are slightly displaced to the left) are plotted; circles are the 10–12 month old mice and hexagons are 15–18 month old mice. The grey line in each panel represents 2SD below the mean for WT mice.

The electroretinogram (ERG) was used to assess retinal function in *C1qtnf5* Ser163Arg KI mice at 10–12 and 15–18 months of age (Figure 6). Representative ERG responses from 18-month-old mutant mice are compared to responses from a wild-type control in Figure 6A. Maximal rod photoreceptor function was elicited with bright light stimuli in the dark-adapted state (Figure 6A, black traces). Cone function was obtained in dark-adapted mice by recording ERGs within a short interval following rod-suppressing bleaching lights (Figure 6A, bottom traces). Delayed dark adaptation following a photobleach, a prominent feature in human L-ORMD [1], [2], was studied in the mice by determining the recovery of the rod ERG photoresponse (a-wave) following a light exposure that completely suppressed it (Figure 6A, gray traces overlapping the DA rod response). ERG responses in mutant animals are similar to those in the wild-type control. ERG a-waves and b-waves were measured conventionally for each of the ERG responses and summary statistics for each group shown (Figure 6B). Rod a-wave amplitudes in homozygous (mean ± SD = 250±53 µV) and heterozygous (238±35 µV) *C1qtnf5* Ser163Arg KI mice were similar to each other and to wild-type (276±56 µV) mice (*P*>0.05). Cone b-wave amplitudes in homozygous (122±23 µV) and heterozygous (109±20 µV) *C1qtnf5* Ser163Arg KI animals were also similar to each other and to wild-type (115±23 µV) mice (*P*>0.05). Recovery of the a-wave (expressed as a fraction of dark-adapted results) in both homozygotes (0.38±0.08) and heterozygotes (0.45±0.10) reached levels comparable to those of wild-type (0.43±0.09) mice (*P*>0.05).

### Effects of photocoagulation/laser induced choroidal neovascularisation

**Figure 7:**
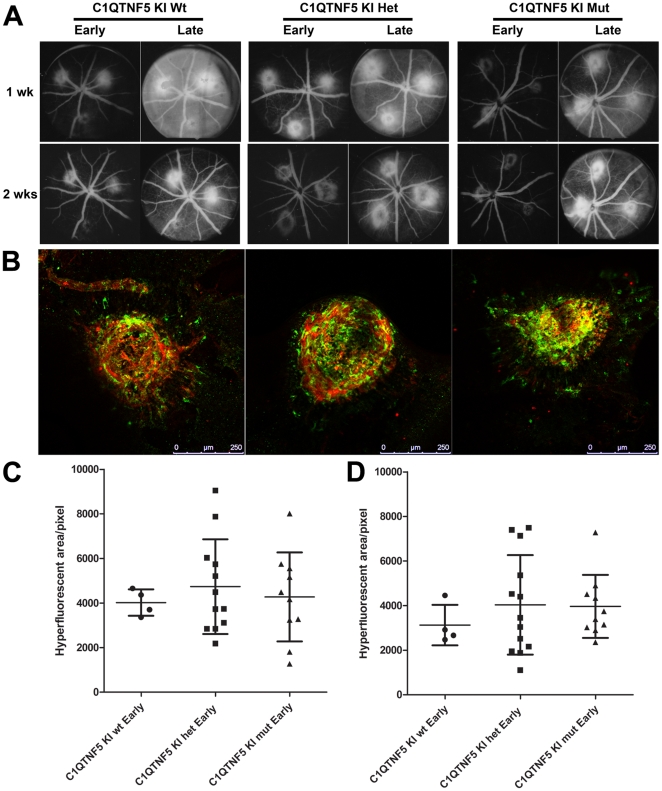
Laser-induced choroidal neovascularization in wild-type and *C1qtnf5* Ser163 Arg knock-in mice. **A**, Fundus images from the early and late phase of *in vivo* fundus fluorescein angiography at 1 and 2 weeks after photocoagulation/laser induced choroidal neovascularization (CNV) in 15–16 month old wild-type and *C1qtnf5* KI mice. Wt: wild-type mice, Het: heterozygous KI mice, Mut: homozygous KI mice. **B**, The microglia in laser lesions on RPE flat mounts resulting from CNV laser treated *C1qtnf5* KI mice after 2 weeks were labelled with anti-Iba1 (green) and co-labelled with anti-lectin (blood vessels) antibodies. Scale bar, 250 µm. **C**,**D**, Quantitative analysis of area of hyperfluorescence in the fundus images from 1 week old animals (**C**) and 2 week old animals (**D**) as a measure for CNV lesion size. No significant difference between the hyperfluorescent areas were observed at 1 week (Kruskal-Wallis with Dunn's mutliple comparison test, p = 0.911) or 2 weeks (Kruskal-Wallis with Dunn's mutliple comparison test, p = 0.638). The number of animals for both time points were n (wt) = 4, n (het) = 12, n(mut) = 10.

Wild-type and heterozygous or homozygous KI mice at 15–16 month of age were exposed to photocoagulation/laser-induced CNV. The results were analysed by *in vivo* fluorescein angiography and immunohistochemistry. There was no obvious difference between the CNV laser lesions at 1 and 2 weeks post-laser in wild-type mice compared to those in *C1qtnf5* Ser163Arg KI mice (Figure 7A). To evaluate the response of microglial cells to laser-induced CNV, retinal and RPE/choroidal flat mounts from laser injured wild-type and *C1qtnf5* KI mice were stained with anti-Iba1 antibody for labelling of microglia. There was no significant difference in microglial cells in the laser lesion area between wild-type and *C1qtnf5* Ser163Arg KI mice (Figure 7B).

### Effects of human mutant *C1QTNF5* over-expression in mouse retina

**Figure 8:**
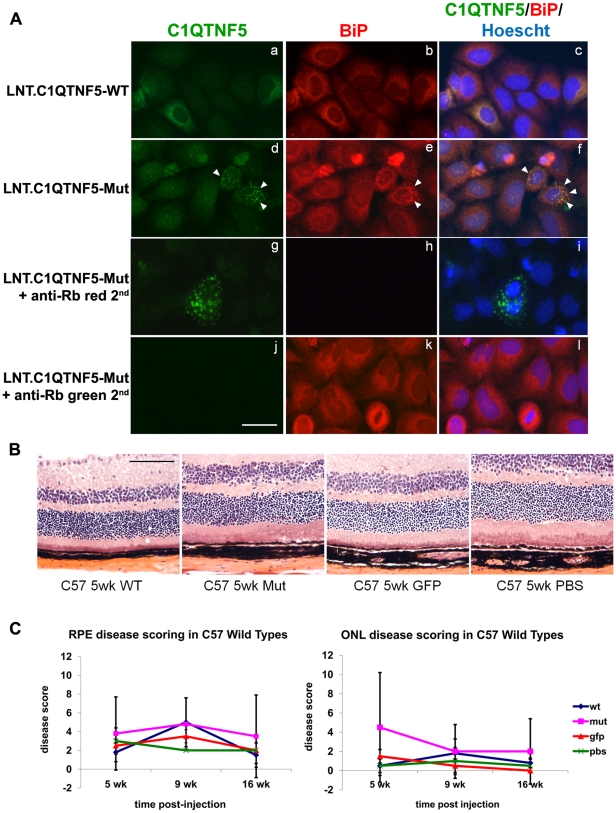
Effects of Lentiviral expression of wild-type or Ser163Arg mutant *C1qtnf5* in mouse retinas. (**A**) HeLa cells were infected with lentivirus contain wild-type C1QTNF5 (LNT.C1QTNF5-WT) or Ser163Arg mutant C1QTNF5 (LNT.C1QTNF5-Mut). At 72 hr post-infection, cells were stained with anti-CTRP5 (green) and the endoplasmic reticulum (ER) marker anti-BiP (red) antibodies. Cells infected with LNT.CTRP5 or LNT. CTRP5-Mut both showed intracellular staining surrounding the nucleus (a, d) suggesting that both proteins enter the secretory pathway. While the staining on LNT-CTRP5 infected cells is rather diffuse (a), the LNT-CTRP5-Mut showed punctate staining (d) that co-localised with the ER marker BiP (e, f, shown by white arrow heads) suggesting that the mutant form of the protein is retained in the cell and cannot exit the ER correctly. Controls showed that there was no contribution to the fluorescent signals from the respective anti-rabbit secondary antibodies (g, h, i, j, k, l). Scale bar = 20 µm. (**B**) Haematoxylin and eosin stained retinal sections from C57BL/6 mouse eyes at 5 weeks after subretinal injection of either LNT.C1QTNF5-WT, LNT.C1QTNF5-Mut, LNT.GFP or PBS. No obvious differences could be observed between the four groups despite some injection related trauma (not shown here). (**C**) RPE and outer nuclear layer (ONL) disease scoring was assessed on randomized images and showed no significant difference between the any of the four treated groups, n = 4 per group (One-way ANOVA, all p-values p>0.05). Scale bar = 100 µm.

We generated lentivirus to over-express human *C1QTNF5* wild-type and Ser163Arg mutant proteins. Initial *in vitro* infection of HeLa cells showed that the wild-type protein was diffusely located in the cytoplasm but most of the mutant protein was retained in the endoplasm reticulum (Figure 8A), consistent with previous observations when *C1qtnf5* is over-expressed [8]. Over-expression of wild-type and mutant C1QTNF5 in wild-type mouse retina did not cause any pathological effects on either RPE or photoreceptors (Figure 8B, C).

## Discussion

Mouse models of human inherited retinal diseases have proved to be invaluable tools for the analysis of disease mechanisms and, more recently, for evaluating therapy [14], [15]. The situation with late-onset or genetically complex disorders, such as age-related macular degeneration, has been less clear-cut due to the presence of anatomical differences (e.g. the mouse has no macula) and the requirement for ageing in addition to inherited and environmental influences, such as diet, genetic background or light exposure. All of these factors may influence both ageing and inflammatory processes, making these disorders intrinsically more difficult to model [16], [17]. Some mouse models of inherited macular dystrophies, including the *Abca4* model of Stargardt disease, reproduce the biochemical features of the human disease but only manifest a very slow photoreceptor degeneration, in contrast to the human disease [18], [19]. A transgenic mouse model of Stargardt-like macular degeneration resulting from *ELOVL4* mutation on the other hand showed both biochemical and functional features of the human disease [20]. Other macular disease models show few features of the corresponding human disease. For example, a mouse model of Sorsby fundus dystrophy, with a *Timp3* Ser156Cys knock-in mutation, showed premature age-related changes in Bruch's membrane and RPE, which were evident at 8 months compared with 30 months in wild-type littermates but lacked the most striking features of the human disease, namely massive sub-RPE deposits (resembling those found in L-ORMD), choroidal neovascularization or retinal atrophy [21].

The lack of overt phenotypic abnormality in *C1qtnf5* Ser163Arg knock-in mice up to 24 months is therefore disappointing but by no means unprecedented in view of the mixed success with mouse models of late-onset macular degeneration. The lack of a macula in the mouse seems unlikely to be a problem since L-ORMD becomes a pan-retinal disease in the later stages. Another possible factor is the short lifespan of the mouse compared with humans, in whom it may take 60–70 years to develop the full disease [22]. However, very recently, Chavali *et al.* [23] reported a very similar knock-in mouse model of L-ORMD in which the same Ser163Arg mutation had been introduced. In contrast to the present study, these mice showed most of the key features of L-ORMD - ERG changes consistent with early dark adaptation abnormalities, accumulation of hyperautofluorescent spots, RPE and Bruch's membrane abnormalities, drusen, loss of photoreceptors and retinal vascular leakage. The Chavali *et al.* [23] mouse was made using a similar targeting construct containing introduced LoxP sites (for subsequently making a *C1qtnf5* deletion knockout) and FRT sites flanking the neomycin selection cassette but these, together with the S163R mutation, were introduced into a bacterial artificial chromosome (BAC) containing the *Mfrp* and *C1qtnf5* genes rather than our approach in which right and left arms were PCR amplified and cloned separately into a plasmid construct and the mutation introduced by standard site directed mutagenesis. In our study, expression of both *Mfrp* and *C1qtnf5* were found to be unaffected in the resultant knock-in mice compared with wildtype (Figure 2). The expression of C1qtnf5 was unaffected in the Chavali *et al.* [23] mouse whereas the expression of *Mfrp* was not checked. One important difference was that Chavali *et al.* targeted C57BL/6J embryonic stem (ES) cell and maintained the resultant knock-in mice on a 100% C57BL/6J genetic background whereas the present mouse model was constructed using a targeted 129SV ES cell and analysed on a mixed 129SV and C57BL/6J background.

Phenotypic differences in mouse models of human disease are commonplace, as are effects of genetic backgrounds (http://phenome.jax.org/) [24]. Many mutations causing human disease show no clinical phenotype in the mouse, even if the targeted protein is completely absent and an appropriate biochemical phenotype is present(e.g. Lesch-Nyhan syndrome, Lowe syndrome, X-linked adrenoleukodystrophy, Fabry disease, galactosemia, Tay-Sachs disease) [25]. Others show mild or partial phenotypes which vary according to the genetic background, including cystic fibrosis, cardiomyopathy, Huntington disease, adenomatous polyposis, glomerulonephritis, Marfan syndrome and hypertension [25], [26], [27], [28], [29], [30], [31], [32], [33]. In some cases, the genetic background differences led to the direct identification of modifier genes [34], [35] while in others, multiple genes appeared to be involved [36]. Phenotypic differences in ocular mutants on different genetic backgrounds are also common, including abnormities of ocular development, early and late onset forms of retinal degeneration and cataract [37], [38] [39], [40] [41]. Again, direct identification of disease modifiers such as variation at the *Rpe65* locus, has proved useful [42]. It remains possible that the phenotypic differences between the *C1qtnf5* knockout mice reported here and by Chavali *et al.* [23] result from differences in the targeting construct, which might for example affect the expression level of the mutant (or wild-type) allele, but an equally likely interpretation is the difference in genetic background. Further work is therefore required to clarify this difference.

Finally, over-expression of the wild-type and mutated forms of C1QTNF5 did not appear to result in any greater RPE or outer nuclear layer pathology than was observed following injection with the control lentiviral construct LNT-GFP or injection with PBS. This suggests, at least over the time points studied, that the mutated protein does not exert a gain-of-function pathogenic effect on either the RPE or ONL, although it is possible that if much longer time points post-injection were studied, a subtle phenotype might become apparent. It remains unclear whether L-ORMD results from a gain-of-function or loss-of-function although over-expression of C1QTNF5 *in vitro* does lead to ER retention [8], as observed with lentivirus over-expression of the mutant protein in HeLa cells (Figure 8).

Resolving phenotypic differences between our *C1qtnf5* KI mouse and that reported by Chavali *et al.* [23] will be difficult. Firstly, it will require back-crossing both KI models onto C57BL/6J or 129SV strains respectively and then repeating the phenotypic analyses in parallel. If the difference is associated with genetic background, it may have therapeutic implications but resolving the mechanism will not be easy since it may be due to polygenic factors influencing penetrance. *In vitro* over-expression of the mutant protein in mouse embryonic fibroblasts from potentially susceptible compared with non-susceptible strains could help to clarify whether genetic background effects are due to differences in the cellular handling of misfolded proteins, but this may vary between tissues, and many alternative explanations are possible.

## Materials and Methods

### Ethics statement

All the animal experiments were performed in accordance with the ARVO Statement for the Use of Animals in Ophthalmic and Vision Research. Animal work was carried out with the approval of the Home Office under project licence number PL 6003868.

### Generation of *C1qtnf5* Ser163Arg knock-in mice

The *C1QTNF5* and *MFRP* genes are located immediately adjacent to one another in both human and mouse genomes and are expressed as a dicistronic transcript [1], [4]. The mouse *Mfrp/C1qtnf5* gene is located on chromosome 9 (position 44204272-44211776) and extends for 7.5 kb. The *Mfrp/C1qtnf5* gene is composed of 14 exons which are expressed in a 4.22 kb bicistronic mRNA. The mouse Mfrp protein is encoded by exons 1 to 13, while the C1qtnf5 protein is encoded by exons 13 and 14 (Figure 1A). We employed a classical approach of homologous recombination to replace wild-type *C1qtnf5* with a modified *C1qtnf5* allele. In order to make the knock-in targeting construct, three genomic fragments were PCR amplified using 129SV genomic DNA as template: the long (5′) homology arm which contained exons 7 to 13 and part of exon 14; the short (3′) homology arm containing the 3′ end of exon 14 and the region located downstream of the *Mfrp*/*C1qtnf5* gene; and a central fragment, containing a portion of exon 14. The Ser163Arg mutation (base change AGC to AGG) in *C1qtnf5* exon 14 was then introduced by site-directed mutagenesis into the central fragment (Figure 1). The above three PCR fragments were cloned into the targeting vector G139 (provided by genOway, Lyon, France) and the linearized construct (Figure 1) was transfected into mouse 129SV ES cells according to genOway's standard electroporation procedures (i.e. 5×10^6^ ES cells in presence of 40 µg of linearized plasmid, 260volt, 500 µF). Positive selection was started 48 hours after electroporation by addition of 200 µg/ml of G418. G418-resistant clones were screened for the correct homologous recombination event by PCR and southern blotting. Three ES clones were used to inject into C57BL/6J blastocysts and a total of 19 chimeric mice were produced from the three injection sessions. Two highly chimeric males (displaying 85% and 98% chimerism) were each mated with two Flp-deleter C57BL/6J females to get heterozygous mice carrying the mutated *C1qtnf5* allele devoid of the neo selection cassette. Agouti F1 progeny were screened for assessing the Flp-mediated excision event of the neo cassette on the *C1qtnf5* targeted allele by PCR and southern blotting, using genomic DNA isolated from tail biopsies (Figure 1C, E). As there was mosaicism for the Flp *C1qtnf5* allele in the F1 mice, the mosaic animals were then crossed with wild-type C57BL/6J mice to generate a pure line of Flp excised heterozygous *C1qtnf5* knock-in mice. The resultant germline *C1qtnf5* Ser163Arg mutant mice were then inter-crossed to produce stocks of heterozygous and homozygous mice on a mixed 129Sv and C57BL/6J genetic background. Genotyping was performed by PCR amplification of a 432 nucleotide base pair (bp) fragment corresponding to the wild-type allele and a 548 bp fragment for the mutant (Ser163Arg) allele using the following primers:-

Forward primer WRI1-F; GAGATGAGTAGTTCCTGAGATGACCACAAGG

Reverse primer WRI1-R; TTGGCAAACCTGGGTGTGTTATCG

The annealing temperature was 65°C (30 secs) and amplification was continued for 35 cycles.

For *in vivo* procedures, the mice were anesthetized by a single intraperitoneal (i.p.) injection of a mixture of medetomidine hydrochloride (1 mg/kg body weight; Domitor; Pfizer Animal Health, New York, NY), and ketamine (60 mg/kg body weight) in water. Whenever necessary, the pupils were dilated with 1 drop of 1% tropicamide.

### Autofluorescence Scanning Laser Ophthalmoscopy (AF-SLO) and semi-quantitative assessment of background autofluorescence

Autofluorescence imaging was performed using a HRA2 scanning laser ophthalmoscope with a 55° angle lense as described previously [13]. We used the autofluorescent mode of the HRA2 to scan the retina with a 488 nm laser that provides the excitation light to stimulate emission of autofluorescence from any possible fluorophores in the retina, including for example lipofuscin in the RPE, which will then be detected by a camera and result in the autofluorescent photographs shown [8]. These photographs are taken systematically withe the optic disc positioned at the centre of the image and the image is focussed either on the inner retinal vasculature or the outer retina respectively. Projection images of 30 frames per fundus are shown and were visually evaluated for brighter spots or areas in the image which would indicate the appearance of autofluorescence. Additionally, in an attempt to semi-quantitatively evaluate the diffuse background autofluorescence of the AF-SLO fundus photographs, all images were converted into 16-bit greyscale images and Image J software was used to analyse the area of pixels above a set threshold for all images. The mean of AF-pixel per fundus photograph above the threshold was calculated for each animal (n = 3 per genotype) using the values obtained for the right and the left eye.

### Electroretinography (ERG)

ERGs were recorded according to previous methodology [43]. Briefly, *C1qtnf5* Ser163Arg KI (n = 12; 6 heterozygotes and 6 homozygotes) mice and age-matched wild-type mice (n = 25) were dark adapted (>12 hours) and anesthetized with ketamine HCl (65 mg/kg) and xylazine (5 mg/kg) intramuscularly under dim red light. Pupils were dilated with 1% tropicamide and 2.5% phenylephrine. Medium energy (10-µs duration) and high energy (1-ms duration) flash stimulators with unattenuated luminances of 0.8 and 3.6 log scot-cd · s · m^−2^, respectively, were used. Neutral density (Wratten 96; Eastman Kodak Company, Rochester, NY) and blue (Wratten 47A; Eastman Kodak Company) filters served to attenuate and spectrally shape the stimuli. Dark-adapted ERGs were obtained with a single blue 2.2 log scot-cd · s · m^−2^ flash. After a 2 minute wait, a series of three white flashes (3.6 log scot-cd.s.m^−2^) were presented in the dark at ∼5-sec intervals which completely suppresses the murine, rod-dominated ERG a-wave. The bright blue stimulus was presented again within 30 seconds of this bleaching light and this elicits an ERG dominated by a cone-mediated b-wave. Next, the extent of recovery of the dark-adapted (DA) rod mediated a-wave was assessed 10 minutes later by recording a further response to the bright blue stimulus. The b-wave amplitudes were measured conventionally, from baseline or a-wave trough to positive peak; a-wave amplitudes were measured from baseline to the trough of the a-wave.

### Laser-induced choroidal neovascularisation (CNV)

Laser-induced CNV and quantification with fundus fluorescein angiography (FFA) were performed as described previously [44]. Three laser lesions per eye were delivered two to three disc diameters from the papillae with a slit lamp–mounted diode laser system (wavelength 680 nm; laser settings: 210-mW power, 100-ms duration, and 100-µm spot diameter; Keeler, Ltd., Windsor, UK). After 1 and 2 weeks, *in vivo* FFA was performed by i.p. injection of 200 µl of 2% fluorescein in PBS and imaging of the fundus at 90 seconds (early) and 7 min (late phase) after fluorescein injection using a small animal fundus camera with appropriate filters (Kowa Genesis,Tokyo, Japan). The pixel area of CNV-associated hyperfluorescence was quantified for each lesion using image-analysis software (Image J, US National Institutes of Health, Bethesda, Maryland, USA, http://rsb.info.nih.gov/ij/). Endothelial cells were labelled with TRITC-conjugated lectin BS-I (*Bandeirea simplicifolia* agglutinin, BS-I) at a 1∶10 dilution (final concentration = 0.1mg/ml, L5264 Sigma Aldrich, Steinheim, Germany).

### Histopathology

Semithin and ultrastructural analyses were performed as previously described [13]. Briefly, eyes were enucleated and fixed in 3% glutaraldehyde and 1% paraformaldehyde in 0.08 M sodium cacodylate-HCl (pH 7.4) for at least 30 hours at 4°C. The cornea and lens were removed and the eye cups oriented and postfixed in 1% aqueous osmium tetroxide for 2 hours, dehydrated by an ascending ethanol series (50%–100%) and propylene oxide, and infiltrated overnight with a 1∶1 mixture of propylene oxide. After 8 hours in full resin, the eyes were embedded in fresh resin and incubated overnight at 60°C. Semithin (0.7 µm) and ultrathin (70 nm) sections were cut in the inferior–superior axis passing through the optic nerve head with a microtome (Ultracut S; Leica, Wetzlar, Germany). Semithin sections were stained with a 1% mixture of toluidine blue-borax in 50% ethanol and images taken using brightfield microscopy (Oberserver.Z1 Axio, Carl Zeiss Microimaging, Jena, Germany). Ultrathin sections sequentially contrasted with saturated ethanolic uranyl acetate and lead citrate for imaging in a transmission electron microscope (TEM model 1010; JEOL, Tokyo Japan) operating at 80 kV. Images were captured with a CCD camera calibrated against a 2160-lines/mm grating (Gatan Orius; Agar Scientific, Stansted, UK) in digital micrograph format and exported as .tiff files into Image J for quantification (Image J, US National Institutes of Health, Behtesda, Maryland, USA, http://rsb.info.nih.gov/ij/). The thickness of Bruch's membrane (BM), defined from the basal lamina of the RPE cell to the basal lamina of the choroidal vessel, was measured in a uniform, randomized way to prevent a potential bias in the observer's choice. The average BM thickness in each eye was obtained from single images taken at a standard magnification of 5000, positioned approximately 1 mm on each side of the optic nerve. After the calibration of Image J, BM was rotated to the horizontal or vertical, and a grid was superimposed on the image. Ten measurements were taken at points where grid lines passed across BM to yield an average value. Finally, the average measurements for superior and inferior field were added, and a mean value ± SD was obtained for BM thickness in each animal.

### Pathology grading

To evaluate pathological effects of the Ser163Arg allele in *C1qtnf5* knock-in mice between 6 and 24 months of age and in lentivirus treated C57BL/6 mice between 5 and 16 weeks post injection, we assessed the respective sections (semithins for the knock-in line and cryosections for the lentivirus treated mice) under bright field microscopy using a 100× objective and a 10× ocular lens, and counted the number of separate individual pathological events, such as cell lysis, pyknosis, swelling, thinning, proliferation and thickening of RPE from the superior to the inferior end of the retina, in each section. From the individual sums of damage per section we calculated the mean sum of RPE alterations from three sections per animal to obtain a representative measure of the RPE damage score per animal, according to a scheme described by Hollyfield *et al.* [12] that we have also used in previous studies [13]. Similarly, we evaluated retinal damage by counting the drop-out of cells from the outer nuclear layer (ONL) towards the RPE, and thinning of the ONL, to assess retinal damage in virus treated eyes. This provided a mean disease score for the ONL of the retina. For the virus-injected eyes, the area directly around the injection sites was excluded to prevent injection-related damage which might influence the RPE or ONL damage scores.

### Immunocytochemistry

8×10^4^ HeLa cells were plated in DMEM+10% FCS+antibiotic/antimycotic (D10) in 96-well plates and incubated overnight. On the following day, cells were infected with either LNT.CTRP5-WT or LNT.CTRP5-Mut in D10. At 72 h post-infection, cells well fixed with ice-cold methanol, washed with PBS containing 0.05% Tween-20 (PBS-T). Cells were blocked with 3% BSA in PBS-T for 1 hr at room temperature (RT) before they were incubated with rabbit-anti-CTRP5 (1∶250 in block solution, antibody provided by A.F. Wright [8]) for 1 hr at RT°C. Cells were washed 4 times in PBS-T and then incubated with goat anti-rabbit-Alexa488 (1∶400 in block, Invitrogen, Paisley, UK) for 45 minutes at RT. For double labelling with the ER-marker BiP, the cells were then washed 4 times in PBS and re-blocked for 1 hr before incubation with the rabbit anti-BiP (1∶100 in block, G8918, Sigma, Gillingham, UK) antibody for 1 hr at RT. After 4 additional wash steps, the goat anti-rabbit-Alexa546 antibody (1∶400 in block, Invitrogen, Paisley, UK) was applied for 45 mins, the cells washed again 4 times before they were counterstained with Hoechst (1∶1000 in PBS-T) and washed once more. For imaging, the wells were kept hydrated with blocking solution in the 96-well plate and imaged with Q-Capture software (Q Imaging, Maidenhead, UK) on an inverted Axio Oberver.Z1 microscope (Carl Zeiss, Jena, Germany).

### Reverse transcriptase-polymerase chain reaction (RT-PCR)

Total RNAs from mouse eye cup tissues were extracted using RNAeasy Mini kit (QIAGEN) according to the manufacturer's instructions. Two micrograms of the resulting RNA was reverse transcribed with random primers using a Transcriptor High Fidelity cDNA Synthesis Kit (Roche). The cDNAs of *C1qtnf5* and *Mfrp* were then amplified using platinum Taq DNA polymerase (Invitrogen) as previous described [45]. PCR primers were manufactured by Sigma Aldrich. Primer sequences are available on request.

### Western blotting

Eyecups from wild-type, heterozygous and homozygous *C1qtnf5* Ser163Arg knock-in mice were lysed in a buffer containing 50 mM Tris-Cl pH 7.5, 150 mM NaCl, 1% NP-40, 0.5% deoxycholate, 0.05% SDS, 2 mM EDTA, 1 mM sodium-vanadate, 5 mM sodium-fluoride and 10 mM iodoacetamide with a protease inhibitor cocktail (Roche). The lysates were centrifuged for 10 min at 13,000 rpm and the post-nuclear supernatants were collected. Equal amounts of protein were electrophoresed on 10% SDS-polyacrylamide gels and transferred to a nitrocellulose membrane. The primary antibodies were used at 1∶1,000 dilution for anti-C1QTNF5 and 1∶2,000 dilution for antiβ-tubulin antibodies. HRP-conjugated secondary antibodies were used at 1∶5,000 dilution. The membrane-bound antibodies were detected by ECL (Amersham Biosciences).

### Lentivirus production

Second generation HIV-1-based self-inactivating lentiviral vectors pseudotyped with the vesicular stomatitis virus glycoprotein envelope contained either the murine *C1qtnf5* wild type or the murine *C1qtnf5* Ser163Arg mutant cDNA or the GFP reporter gene (Lenti.GFP). All vectors contain deletions in the 3′ long-terminal repeats, making them self-inactivating, and include the central polypurine tract (cPPT), which is necessary for second-strand DNA synthesis, and may enhance the nuclear transport of the provirus. All cDNAs are driven by the spleen focus-forming virus (SFFV) promoter and each vector contains the woodchuck hepatitis virus post-transcriptional regulatory element (WPRE) to stabilize mRNA and increase expression. Vectors were produced using transient triple transfection of 293T cells as described previously [46]. At 28–72 hr post-transfection, supernatant was harvested and filtered through a 0.45 µm filter. The virus particles were concentrated by ultracentrifugation at 90,000 g for 2 hr and the pelleted virus was resuspended in OptiMEM and stored at −80°C. Virus was titred using a Reverse Transcriptase activity ELISA kit (Roche Diagnostics, Burgess Hill, UK) according to the manufacturer's instructions. Transgene expression was detected by immunocytochemistry on infected HeLa cells. Cells were infected with 1 µl of virus in a 24 well plate. Three days post-infection, cells were washed and fixed with ice-cold methanol and stained with a polyclonal rabbit anti-human C1QTNF5 antibody [8] and goat anti-rabbit AlexaFluor546 antibody. Cells were counterstained with Hoechst, imaged using a Zeiss Axioplan II fluorescent microscope and analysed using QCapture Pro software (QIMaging, Maidenhead,UK).

### Subretinal injections

Subretinal administration of vectors was performed in anesthetized 12 to 16-week-old C57BL/6 mice (Harlan UK Ltd.), under direct retinoscopy through an operating microscope as previously described [47]. A 1.5-cm 34-gauge hypodermic needle (Hamilton, UK) was used to inject 2 microlitres of virus suspension (titer of 10^8^ particles/ml) into the subretinal space to produce a bullous retinal detachment in the superior hemisphere and a second in the inferior hemisphere. Mice received *C1qtnf5*-wild type virus in one eye and *C1qtnf5*-Ser163Arg mutant virus in the other eye. Control mice received PBS or titre-matched Lenti.GFP. Retinal vessels remained patent during and following the injections. All animals received chloramphenicol 1% eye ointment to the cornea.

### Statistical analysis

Statistical analyses were performed using GraphPad Prism 5 for Windows (GraphPad Software Inc, La Jolla, USA). Changes in the thickness of Bruch's membrane or in RPE damage with age were assessed using Pearson correlation statistics, a parametric measure of association for two continuous random variables. Differences in autofluorsecence were assessed using a Kruskal-Wallis with Dunn's multiple comparison test. RPE and outer nuclear layer disease scoring was assessed on randomized images using a one-way analysis of variance.
